# Estimation of Leaf Phosphorus Content in Cotton Using Fractional Order Differentially Optimized Spectral Indices

**DOI:** 10.3390/plants14101457

**Published:** 2025-05-13

**Authors:** Mamat Sawut, Xin Hu, Yierxiati Abulaiti, Rebiya Yimaer, Baidengsha Maimaitiaili, Shanshan Liu, Ran Pang

**Affiliations:** 1 College of Geography and Remote Sensing Sciences, Xinjiang University, Urumqi 830017, China; 107552201144@stu.xju.edu.cn (X.H.); rby217@xju.edu.cn (R.Y.); lss20001124@163.com (S.L.); pangran_157@163.com (R.P.); 2 Xinjiang Key Laboratory of Oasis Ecology, Xinjiang University, Urumqi 830017, China; 3 Key Laboratory of Smart City and Environment Modelling of Higher Education Institute, Xinjiang University, Urumqi 830017, China; 4 Xinjiang Field Scientific Observation and Research Station for the Oasisization Process in the Hinterland of the Taklamakan Desert, Xinjiang University, Hotan 848400, China; 5 National Cotton Engineering Technology Research Center/Cotton Research Institute of Xinjiang Uyghur Autonomous Region Academy of Agricultural Sciences, Urumqi 830091, China; yierxiati@xaas.ac.cn (Y.A.); 107552201151@stu.xju.edu.cn (B.M.)

**Keywords:** LPC, spectral indices, FOD, random forest

## Abstract

The accurate and timely detection of leaf phosphorus content (LPC) is extremely important for the fertilization management of crop growth and yield. This study aimed to establish an estimating model of LPC in cotton based on hyperspectral data. Under field experimental conditions with different phosphorus treatments, the spectral data and LPC were measured. The spectral characteristics of different cotton cultivars and leaves with varying phosphorus content were analyzed. Optimized spectral indices most correlated to phosphorus were calculated with combinations of arbitrary bands using the Fractional Differential Order (FOD) transform. Then, the random forest-based(RF) estimation model for cotton LPC was established. The research results indicated that (1) the spectral changes of 24 cotton cultivars were basically consistent, and spectral differences between the cultivars became more obvious within the 760–960 nm spectral region; (2) in the visible region, the reflectance of cotton under different phosphorus treatments did not show obvious regularity, while in NIR, the reflectance of cotton increased with the increase in phosphorus content, showing a certain difference in phosphorus; (3) the RF model using a difference spectral index (DSI) had the best performance for LPC estimations in calibration (R^2^ = 0.78) and validation (R^2^ = 0.85), which was superior to the other models based on two spectral indices (the NDSI and RSI). This study provides technical support for the hyperspectral estimation of LPC in cotton.

## 1. Introduction

Phosphorus is one of the essential minerals that plants need to grow and yield [1]. An adequate amount of phosphorus increases plant water use efficiency, improves the efficiency of other nutrients, such as nitrogen, contributes to disease resistance in some plants, helps plants cope with cold temperatures and moisture stress, hastens plant maturity, and protects the environment through better plant growth [2]. Effective diagnosis of crop phosphorus content plays an important role in precise phosphorus management and the improvement of phosphorus fertilizer use efficiency. Traditional methods for the determination of phosphorus content usually rely on laboratory chemical analysis, which is accurate but time-consuming and labor-intensive, and it is difficult to meet the needs of rapid and nondestructive monitoring in precision agriculture [3].

The rapid development and expanded application of hyperspectral remote sensing technology have provided advanced technical means for the real-time, quantitative, and rapid monitoring of phosphorus content in crops [4]. Understanding the spectral mechanisms of crop leaves forms the foundation of quantitative phosphorus estimations. The relationship between spectral reflectance and phosphorus status has been investigated. Osborne et al. [5] identified distinctive spectral features in phosphorus-deficient maize within blue (440–445 nm) and near-infrared (730–930 nm) regions. Ban et al. [6] analyzed the spectral characteristics of phosphorus content in rice leaves from UAV hyperspectral images and observed inverse correlations between rice canopy reflectance and phosphorus content at visible (454–720 nm) wavelengths, while no significant patterns emerged at NIR (724–998 nm) wavelengths. Zhang et al. [7] found that the trend of the spectral reflectance curve of maize leaves was roughly the same at different growth stages, and it was reported that with the increase in phosphorus fertilizer application, the spectral reflectance of maize leaves showed an upward trend, especially the NIR band. These foundational studies demonstrate the spectral potential for phosphorus monitoring in various crops, but there are also many inconsistencies in the research results. These inconsistencies are influenced by a variety of factors, such as diverse crop types, varying growing stages, and external environmental conditions.

Recent research has focused on optimizing spectral data processing and modeling through multi-band combinations and different machine learning integrations. Mahajan et al. [8,9] studied the correlation between spectral indices (SI) and LPCs for wheat and rice, analyzing the differences, ratios, and normalized spectral indices formed via VIS–NIR, and proposed optimized spectral indices for the estimation of the LPC in wheat and rice, which was named the dual-band vegetation index ((R_1080nm_/R_1460nm_) and (R_1260nm_ − R_670nm_)/(R_1260nm_ − R_670nm_)). Zhu et al. [10] investigated the relationship between phosphorus content and spectral indices in apple flowers and found that SIs such as the DVI (936 nm, 676 nm), DVI (977 nm, 676 nm), NDVI (936 nm, 676 nm), and NDVI (977 nm, 676 nm) have a high correlation with the phosphorus content in apple flowers. Among them, the monitoring model constructed with the NDVI had the highest accuracy, with a coefficient of determination R^2^ of 0.94. Unlike the two-dimensional spectral index, other research results showed that the three-band vegetation spectral index can be more effective in estimating the LPC in Bermuda grass [11]. However, the relationship between the phosphorus content in crops and SIs is still inconsistent. To develop an optimized SI and improve the model accuracy, researchers have tried to use mathematical transformations for the original spectrum [12]. Li et al. [13] carried out spectral data prepossessing such as continuous removal, first derivatives, and reciprocal logarithm for the rapeseed canopy spectrum and found that the PLS model with first derivatives has a significantly improved prediction performance than other spectral transformation methods. The coefficients of determination (R^2^) for the calibration dataset and validation dataset are 0.822 and 0.769, respectively. In addition to spectral processing, the selection of modeling algorithms critically impacts prediction accuracy. Therefore, machine learning has been applied to estimate the LPC in crops [14,15]. Chen et al. [16] developed a partial least squares regression (PLSR) model to estimate the LPC in sugarcane leaves, achieving an R^2^ value of 0.99. A support vector machine (SVM) model was created by Gao et al. [17] for the estimation of forage LPC. Wei et al. [18] employed a K-nearest neighbor (KNN), a back-propagation neural network (BP), and a random forest (RF) algorithm to develop models for estimating the LPC in walnut trees, and the results showed that the RF-based model achieved the best performance. Yu et al. [19] applied a whale optimization algorithm (WOA) to optimize extreme learning machine (ELM), used to collaboratively estimate the nitrogen and phosphorus contents of japonica rice, and the results showed that the WOA-ELM model was better than that of the ELM model, with the nitrogen inversions R^2^ up to 0.7045 and the phosphorus inversion R^2^ up to 0.4297. However, previous research has demonstrated significant variability in the performance of different modeling algorithms. Consequently, the careful selection of appropriate input variables is crucial for optimizing the application of machine learning algorithms in the accurate estimation of LPC.

While current research has advanced in identifying sensitive spectral bands and developing estimation models, limitations persist in spectral index consistency and model generalizability. Compared to chlorophyll and nitrogen spectral studies, phosphorus-specific spectral diagnostics research remains underdeveloped, with an insufficient theoretical framework. Therefore, to improve modeling accuracy, an integration of sensitive spectral bands, spectral transformation techniques, and optimized spectral indices (SI) with machine learning algorithms still needs investigation.

This research aimed to (1) systematically analyze spectral reflectance responses to phosphorus treatment in cotton; (2) optimize spectral indices through combinations of any two bands using fractional order differential (FOD) transformation, and (3) develop RF-based hyperspectral estimation models for LPC in cotton.

## 2. Materials and Methods

### 2.1. Research Area and Experimental Design

**Figure 1:**
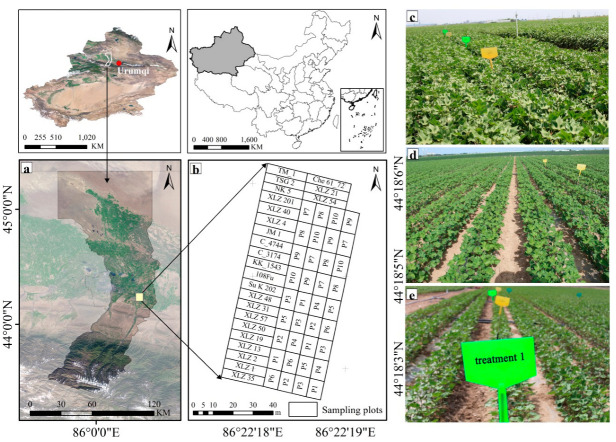
Location of study area and distribution of sampling plots: (**a**) Manas county, (**b**) sampling plots, and (**c**–**e**): photo of experimental field.

The experimental site was situated at the Cotton Germplasm Improvement Experimental Station (85°19′–86°25′ E, 44°16′–44°22′ N) of the Xinjiang Academy of Agricultural Sciences in western China (Figure 1a). The region features a temperate continental climate with an annual mean temperature of 7.2 °C, frost-free periods of 165–172 days, and an average annual precipitation of 173.3 mm (including 74.4 mm snowfall). Annual evapotranspiration ranges from 1500 to 2100 m. Soil analysis of the tillage layer (0–20 cm) revealed the following physicochemical properties: organic matter content 10.0 g·kg^−1^, alkali-hydrolyzable nitrogen 10.3 mg·kg^−1^, available phosphorus 21.9 mg·kg^−1^, available potassium 389.0 mg·kg^−1^, pH 8.22, and electrical conductivity 1.55 mS·cm^−1^, with a silt loam texture [20].

The field experiment on cotton was carried out at the experimental station from April to September 2017. The experimental field was divided into a total of 60 plots. Twenty-four plots were allocated to 24 cotton cultivars that were historically cultivated in Xinjiang between 1950 and 2017, and included the following: Xinluzhong 35 (XLZ 35), Xinluzao 1 (XLZ 1), Xinluzao 2 (XLZ 2), Xinluzao 13 (XLZ 13), Xinluzao 19 (XLZ 19), Xinluzao 50 (XLZ 50), Xinluzao 57 (XLZ 57), Xinluzao 31 (XLZ 31), Xinluzhong 4 (XLZ 4), Xinluzhong 40 (XLZ 40), Xinluzhong 201 (XLZ 201), Xinluzhong 54 (XLZ 54), Xinluzhong 21 (XLZ 21), Xinluzhong 48 (XLZ 48), Su K202, 108Fu, KK-1543, C-3174, C-4744, Junmian 1 (JM1), Nongken 5 (NK5), Tashkent 2 (TSG2), Che61-72, and the genetic standard TM-1. All cultivars received nitrogen fertilization (450 kg·ha^−1^ urea as a top-dressing without phosphorus supplementation.

The remaining 36 plots utilized Xinluzao 57(XLZ 57) to establish ten phosphorus gradient treatments: P1 (straw incorporation + zero phosphorus), P2 (75 kg/hm^−2^ phosphate fertilizer), P3 (150 kg/hm^−2^ phosphate fertilizer), P4 (300 kg/hm^−2^ phosphate fertilizer), P5 (600 kg/hm^−2^ phosphate fertilizer), P6 (1200 kg/hm^−2^ phosphate fertilizer), P7 (no straw + zero phosphorus), P8 (double straw + 82.5 kg/hm^−2^ phosphate fertilizer), P9 (organic fertilizer (containing 150 kg/hm^−2^ phosphate fertilizer)), and P10 (organic fertilizer (containing 300 kg/hm^−2^ phosphate fertilizer)). Each treatment was replicated 3–4 times in a completely randomized arrangement. Seeds were sown on 28 April 2017, followed by emergence irrigation on 5 May. All other agronomic practices followed local cotton production standards. The experimental layout is illustrated in Figure 1b.

### 2.2. Determination of Cotton Leaf Phosphorus Content (LPC)

At each critical growth stage of cotton, a representative sampling area (30 cm × 30 cm) was systematically selected from the central of each experimental plot. Approximately 20 leaves from different plant positions were collected per sampling unit. Fresh samples were immediately stored in sealed polyethylene bags and transported to the laboratory for processing. In the laboratory, the samples were thoroughly cleaned using degreased cotton, followed by a 30 min withering treatment at 105 °C, and then dried to a constant weight at 75 °C. Afterwards, the samples were crushed and sieved, and the leaf total phosphorus content of the cotton leaves was determined using the vanadium molybdenum yellow colorimetric method.

### 2.3. Spectral Data Measurement and Processing

#### 2.3.1. Reflectance Data Acquisition

In this study, cotton spectral measurements were carried out at the same time as the leaf sampling in cotton. The ASD FieldSpec HandHeld portable spectroradiometer (ASD Inc., Boulder, CO, USA) was employed to measure the leaf spectrum. The spectral range of this instrument is from 325 to 1075 nm, with a spectral resolution of up to 3 nm, and it achieved a resolution of 1 nm after resampling. Crops are highly sensitive to phosphorus during the seedling stage. During this period, cotton absorbs the most phosphorus, and its phosphorus content is relatively high. Management during this stage is crucial for the yield and quality of cotton. Therefore, on 15 June 2017, during the seedling stage, spectral measurements were conducted under clear, windless, or low-wind-speed weather conditions. The measurement duration spanned from 11:30 to 15:30. The spectral probe was maintained at a height of 25 cm above the cotton canopy and ensured to be vertically downward facing. The spectral scanning time was set to 8 s, and 6 measurements were taken at each sampling point. A whiteboard calibration was performed after every 3 measurements. Additionally, the original spectra were smoothed using the commonly used Savitzky–Golay filtering method.

#### 2.3.2. Fractional Differential Order

Fractional Differential Order (FOD), as an extension of integer-order differentiation, has demonstrated significant advantages in reducing background noise and enhancing spectral information. Based on this, we employed the Grünwald–Letnikov (G–L) fractional differential equation to process spectral data within the differentiation range of 0 to 2 orders, using a step size of 0.1. The definition of FOD is as follows [21]:

(1) $$d^{\alpha }f(x)=\underset{h\to 0}{lim}\frac{1}{d^{\alpha }}{\sum_{m=0}^{\left[(t-a)/h\right]}\left(-1\right)}^{m}\frac{\Gamma \left(\alpha +m\right)}{m!\Gamma \left(\alpha -m+1\right)}f\left(x-mh\right)$$

In the equation, x represents the corresponding band, α is an arbitrary order, h is the differentiation step size, t and a are the upper and lower limits of differentiation, respectively, and m is the difference between the upper and lower limits of differentiation. Γ denotes the Gamma function. Since the resampling interval of the ASD spectrometer is 1 nm, we set h = 1 in Equation (1). The fractional differential expression for the function f(x) is derived as follows:

(2) $$\frac{d^{v}f\left(x\right)}{{dx}^{v}}\approx f\left(x\right)+\left(-v\right)f\left(x-1\right)+\frac{\left(-v\right)\left(-v+1\right)}{2}f\left(x-2\right)+\ldots \;+\frac{\Gamma \left(-v+1\right)}{m!\Gamma \left(-v+m+1\right)}f\left(x-m\right)$$

In Equation (2), v denotes the order. When v = 0, it corresponds to the original spectrum. When v is an integer, it represents integer-order differentiation, where v = 1 and v = 2 are commonly used for first-order and second-order integer differentiation in spectral processing, respectively.

#### 2.3.3. Optimized Spectral Indices

This study leverages the characteristics of hyperspectral data, which consist of numerous and continuous bands, to employ an arbitrary band combination algorithm, aimed at considering all suitable combinations of the full band. The algorithm calculates the normalized difference spectral index (NDSI), the difference spectral index (DSI), and the ratio spectral index (RSI) of the spectra. The definition of the optimized spectral index is as follows:

(3) $$NDSI=(R_{\lambda_{m}}-R_{\lambda_{n}})/(R_{\lambda_{m}}+R_{\lambda_{n}})$$

(4) $$DSI=(R_{\lambda_{m}}-R_{\lambda_{n}})$$

(5) $$RSI=R_{\lambda_{m}}/R_{\lambda_{n}}$$

In the formula: $\lambda_{m}$ and $\lambda_{n}$ represent any two unequal wavelengths within the range of 325–1075 nm; R is the spectral reflectance corresponding to the wavelength.

### 2.4. Algorithm and Modeling

In this study, Pearson correlation analysis was employed to screen sensitive bands and a selection of the optimized spectral indices. A prediction model for the LPC in cotton was established using the random forest (RF) algorithm with the optimized spectral indices as the independent variable. The stability and predictive ability of the model were validated using the coefficient of determination (R^2^) and root mean square error (RMSE). An R^2^ value closer to 1 indicates stronger stability of the estimation model, while a smaller RMSE value signifies higher prediction accuracy of the model [20,21]. Among the 60 sets of sample data collected, 36 sets (60%) were randomly selected as the calibration dataset, and the remaining 24 sets (40%) were used as the validation dataset. The division of the sample dataset is shown in Table 1.

## 3. Results

### 3.1. Spectral Analysis of Different Cotton Varieties

**Figure 2:**
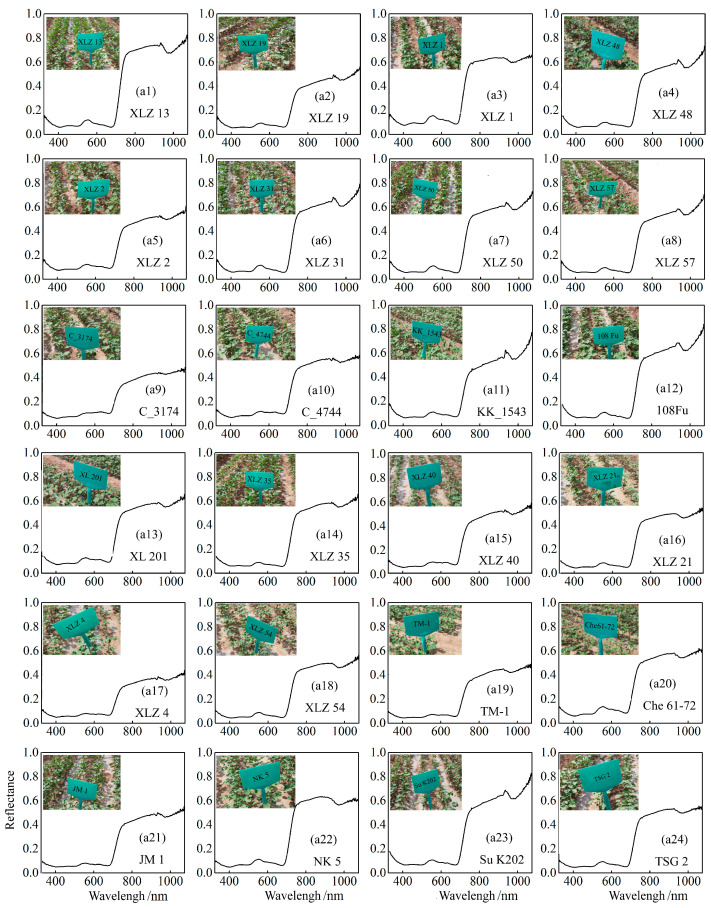
Spectral curves of cotton leaves of 24 different cotton varieties.

Figure 2 shows the spectral reflectance for 24 cotton varieties. As a result, the spectral curves of different cotton varieties generally align with the typical spectral characteristics of green plants. In the visible band (380–760 nm), the spectral reflectance of all varieties is relatively low (approximately 20%), with a smooth curve shape. The spectral curve exhibits an initial upward trend followed by a decline, showing distinct absorption valleys at 460 nm in the blue band and 680 nm in the red band. In the yellow edge region, reflectance gradually decreases, while a prominent chlorophyll-induced reflection peak appears at 540 nm in the green light band. Within the 690–760 nm band, the spectral curve undergoes significant fluctuations, with reflectance sharply increasing as the wavelength rises, demonstrating a notable ‘red edge’ effect. In the NIR beyond 760 nm, the reflectance of the cotton canopy rapidly increases and stabilizes, ranging between 40% and 60%, with an absorption valley observed at 960 nm.

**Figure 3:**
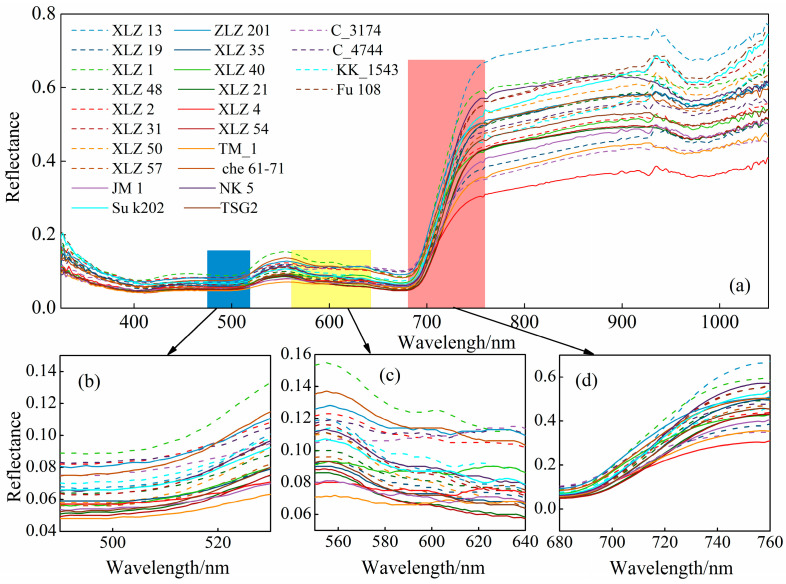
Three edge characteristics of cotton canopy under 24 different types from XinJiang: (**a**) visible and NIR, (**b**) blue edge, (**c**) yellow edge, and (**d**) red edge.

Figure 3 further illustrates the overall variations in spectral reflectance among 24 cotton varieties across the visible–near-infrared (Vis–NIR) band and sensitive regions such as the blue edge, yellow edge, and red edge. As shown in Figure 3a, within the Vis–NIR range (380–1075 nm), the spectral reflectance curve trends of different cotton varieties are almost similar, yet subtle differences exist depending on the variety and specific wavelength. In the visible region (380–760 nm), differences in spectral reflectance among cotton varieties are less distinct. Specifically, in the blue edge (Figure 3b) and yellow edge (Figure 3c) regions, XLZ1 exhibits the highest reflectance, while TM-1 shows the lowest. In contrast, within the red edge region (Figure 3d), XLZ13 displays the highest reflectance, and XLZ4 the lowest. Starting from 760 nm in the NIR region, spectral differences among varieties become more pronounced, particularly within 760–960 nm. In this range, spectral differences among varieties become more pronounced, particularly within 760–960 nm. In this range, the spectral reflectance of cotton varieties generally follows the descending order: XLZ13 > XLZ1 > NK5 > 108Fu > XLZ31 > SuK202 > XLZ50 > XLZ201 > Che6172 > XLZ35 > XLZ48 > C-4744 > XLZ57 > KK-1543 > TSG2 > XLZ2 > XLZ40 > XLZ54 > XLZ21 > M1 > XLZ19 > TM-1 > C-3174 > XLZ4

### 3.2. Spectral Analysis of Cotton Under Different Phosphorus Treatments

**Figure 4:**
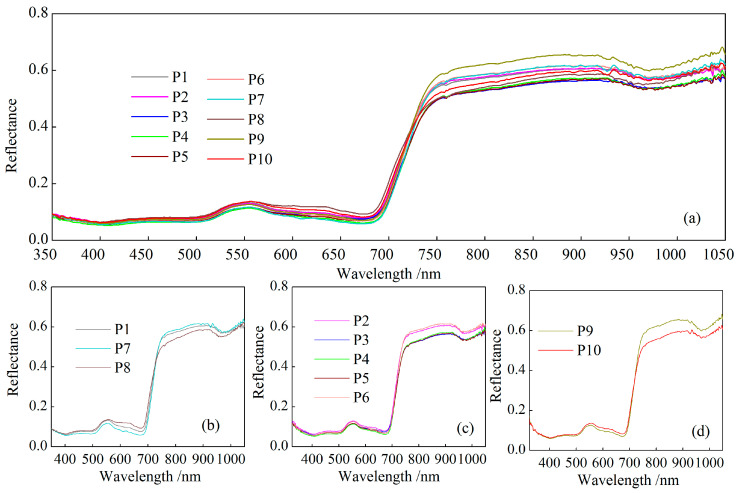
Spectral curve of cotton leaves under different phosphate treatments: (**a**) all 10 gradients, (**b**) straw–phosphorus combinations, (**c**) five phosphorus gradients, and (**d**) organic fertilizer treatments.

To investigate the influence of phosphorus treatment on cotton spectrum, this study classified 10 phosphorus gradients into three groups based on the phosphorus treatment levels. The results are illustrated in Figure 4. Figure 4a displays the spectral curves across all phosphorus gradients. Overall, the phosphorus treatment exerted a measurable influence on the spectral reflectance of cotton leaves, with variations observed across different spectral bands. Compared to the visible band, the spectral reflectance in NIR exhibited an increasing trend with higher phosphorus inputs, accompanied by distinct differences in the spectral response curve. Figure 4b presents the spectral curves under combined straw and phosphorus treatments. In the visible band, spectral reflectance increased with higher straw and phosphorus application rates, a trend similarly observed in the NIR, with the overall order of reflectance intensity being P8 > P1 > P7. Figure 4 illustrates the spectral curves of cotton under five phosphorus gradients. In the visible region, no clear correlation emerged between the reflectance and phosphorus application levels. However, in the NIR region, reflectance initially increased with the phosphorus levels up to P2, following the order P6 > P5 > P4 > P3, but unexpectedly rose under the P2 treatment when phosphorus application was further reduced. Figure 4d shows the spectral curves under organic fertilizer and phosphorus gradients. In the NIR region, leaf spectral reflectance decreased with higher phosphorus inputs (P9 > P10), while the opposite trend was observed in the visible band. These analyses reveal the complexity of cotton spectral responses to phosphorus levels. While spectral differences in the visible region were subtle, a pronounced NIR reflectance plateau emerged in the NIR band, demonstrating gradient-specific variations linked to the phosphorus content. These spectral distinctions provide a foundational basis for estimating LPC through hyperspectral remote sensing.

**Figure 5:**
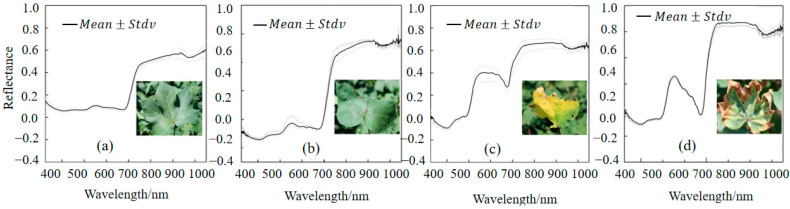
Spectral curves and standard deviations of cotton leaves under different phosphorus stresses: (**a**) health status, (**b**) phosphorus deficiency stage 1, (**c**) phosphorus deficiency stage 2, and (**d**) phosphorus deficiency stage 3.

Figure 5 shows the spectral characteristic curves of cotton leaves under different P stress conditions. As shown in Figure 5, the response of the vegetation spectrum to LPC changes is usually weak under early or moderate phosphorus changes. Only when there is severe phosphorus deficiency or at the end of phosphorus deficiency, and obvious visual symptoms such as a bronze color at the leaf edges, anthocyanin accumulation, or necrosis occur, will more significant characteristic changes occur in the spectrum. Specifically, the spectral characteristics of cotton leaves in a healthy state show relatively stable features. With the decrease in phosphorus content, the fluctuation of the spectral curve of cotton leaves is obvious.

### 3.3. Correlation Between Spectral Parameters and LPC

#### 3.3.1. Correlation Between Spectra and LPC

Table 2 presents the variation in correlation coefficients between LPC in cotton and raw spectral reflectance, as well as fractional order differentiation (FOD) processed spectral reflectance. As shown in Table 2, the significant correlation coefficients between raw spectral reflectance and LPC are primarily distributed within the NIR band. Within the NIR, 151 bands exhibited correlations significant at the 0.01 level, with the maximum absolute correlation coefficient R of 0.422 observed at 973 nm. These results highlight the critical reference value of this specific band for estimating cotton leaf phosphorus content.

The correlation between spectral reflectance and LPC improved by FOD processing. Within the 0–1 order differentiation range, the correlation between differentiated spectra and LPC initially increased and then decreased with higher differentiation orders. The highest absolute R values were concentrated at 459 nm, 838 nm, 940 nm, and 970 nm. The maximum absolute R (0.514) occurred at 940 nm under 0.5 order differentiation. Within the 1–2 order differentiation range, correlations similarly exhibited an initial increase followed by a decline with higher orders, while the number of bands passing significance tests gradually decreased. The highest maximum absolute R values in this range were concentrated at 459 nm and 838 nm, with the maximum absolute R (0.547) observed at 838 nm under 1.6 order differentiation.

**Figure 6:**
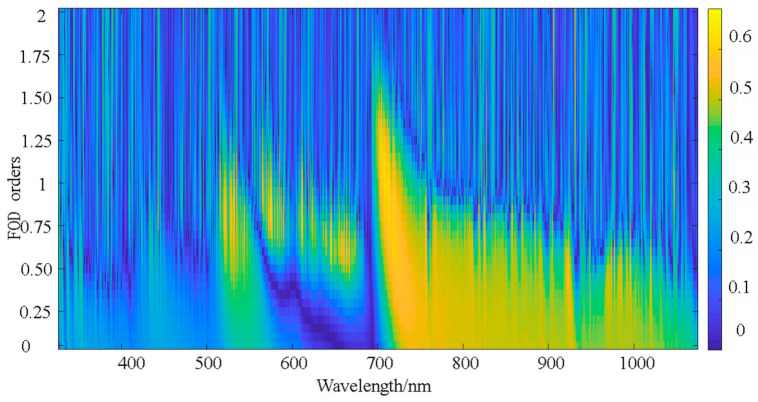
Correlation between cotton phosphorous content and FOD spectra.

Figure 6 further illustrates the distribution of the correlation coefficients across wavelengths for different fractional order spectra, corroborating the aforementioned findings. In summary, the maximum absolute correlation coefficients R peaked in fractional order differentiated spectra. Notably, spectra within the 1.4–1.8 differentiation order range demonstrated heightened sensitivity to phosphorus content.

#### 3.3.2. Correlation Between Optimized Spectral Indices and LPC

Contour maps of the correlation coefficients were generated with the RSI, DSI, and NDSI using raw spectra, as well as frequently used first-order and second-order derivatives and the optimal fractional order derivative (1.6 order) spectra. The results are illustrated in Figure 6.

**Figure 7:**
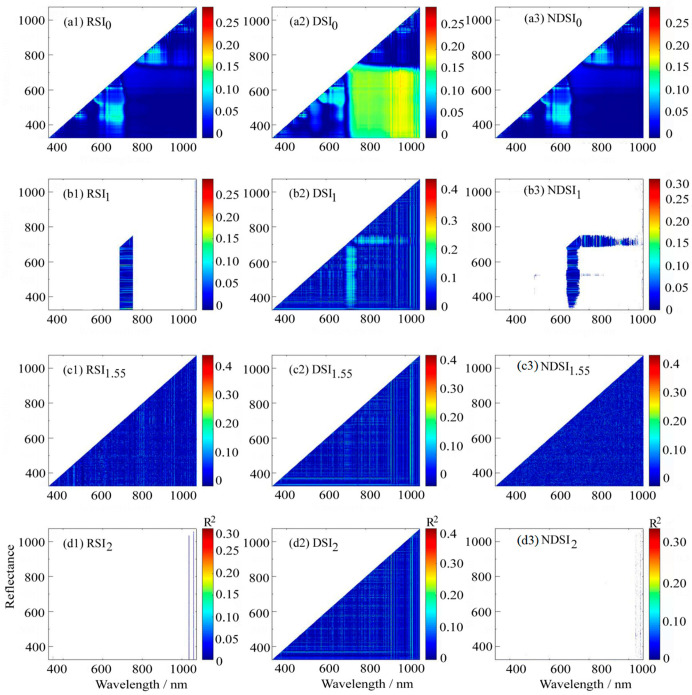
Determination coefficients (R^2^) between LPC and optimized SI.

Figure 7(a1)–(a3) shows that for raw spectral data, the optimized spectral indices sensitive to phosphorus content were RSI (835 nm, 839 nm), DSI (1062 nm, 1065 nm), and NDSI (835 nm, 839 nm), with coefficients of determination (R^2^) of 0.298, 0.295, and 0.298, respectively. Notably, the RSI and NDSI shared identical sensitive bands.

Figure 7(b1)–(b3) demonstrates that after first-order derivative transformation, the optimized spectral indices for phosphorus sensitivity were RSI (617 nm, 1067 nm), DSI (675 nm, 839 nm), and NDSI (1067 nm, 617 nm), yielding R^2^ values of 0.276, 0.425, and 0.3, respectively.

Figure 7(c1)–(c3) presents the results based on 1.6 order, where the optimized spectral indices were RSI (852 nm, 737 nm), DSI (839 nm,452 nm), and NDSI (491 nm,487 nm), with R^2^ values of 0.459, 0.415, and 0.409, respectively.

Figure 7(d1)–(d3) shows that after second-order derivative transformation, the optimized indices were RSI (606 nm, 1060 nm), DSI (839 nm, 452 nm), and NDSI (522 nm, 341 nm), achieving R^2^ values of 0.3, 0.409, and 0.3, respectively.

### 3.4. Estimation Model for LPC Based on Optimized Spectral Indices

Once the SIs were calculated, such as the NDSI, RSI, and DSI, the RF model was developed to estimate the LPC in cotton, and the results are given in Table 3. As can be seen from Table 3, among the three optimized SIs, the DSI-based RF model achieved the highest coefficient of determination (R^2^ = 0.78) and the lowest root mean square error (RMSE = 0.008). In contrast, the model using the RSI exhibited the lowest predictive accuracy, with R^2^ = 0.59 and RMSE = 0.01. The validation results further confirmed the superior performance of the DSI-based model, and the estimation accuracy has been improved, with R^2^ and RMSE values of 0.85 and 0.007, respectively.

**Figure 8:**
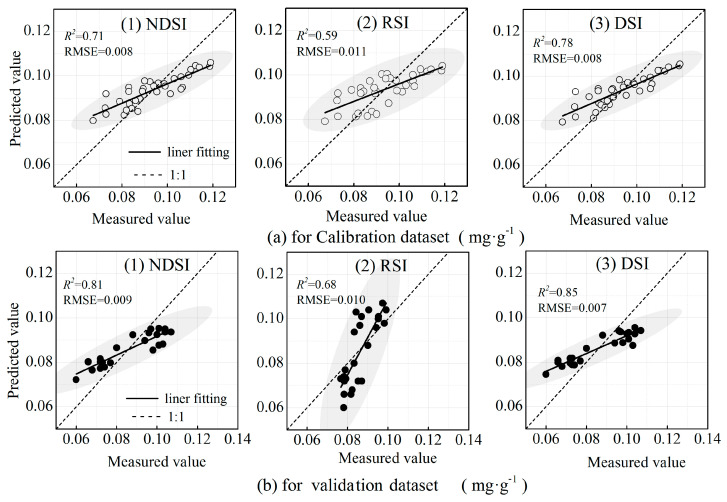
Scatter plot fitting of the measured and predicted values for the total phosphorous content in cotton by RF model.

Scatter plots of the measured and predicted LPCs are illustrated in Figure 8. The DSI-based model showed that the predicted values distributed evenly around the 1:1 reference line for both the calibration and validation datasets, indicating strong agreement with the measured values without obvious overestimation or underestimation. This suggests that the model has higher accuracy in estimating the LPC.

## 4. Discussion

Due to the low phosphorus content in crops, the spectral information is weak. Therefore, establishing the quantitative relationship between crop phosphorus and spectral features, as well as the identification of sensitive bands, is a challenging research area. This study revealed common spectral trends in leaves across diverse cotton varieties in Xinjiang while highlighting significant differences within the NIR band. Furthermore, under varying phosphorus treatments, spectral reflectance in the visible band showed no consistent patterns, whereas NIR spectral reflectance increased with higher phosphorus inputs, demonstrating gradient-specific variations. This phenomenon may be attributed to differences in phosphorus use efficiency and partial productivity under varying phosphorus treatments, which influence leaf pigmentation, morphology, and cellular structure, thereby modulating the NIR spectral responses. Previous studies have similarly emphasized that leaf pigments govern spectral reflectance in the visible range, while the cellular structure dictates reflectance in NIR [18]. Mistele et al. [22] observed the same regularity in predicting LPC in cotton under water–nitrogen coupling conditions, though the specific sensitive bands for phosphorus differed. And he pointed out that under different growth periods and phosphorus treatments, the spectral reflectance of corn showed high consistency, and the spectral reflectance increased with an increase in the phosphorus amount in NIR. These are basically consistent with the results of this study. However, some scholars’ research [23] showed that in the visible band, the spectral reflectance of cotton increases with an increase in phosphorus content, while in NIR, the spectral reflectance decreases with an increase in phosphorus content, and they identified sensitive bands at 760–1300 nm, 1650–1830 nm, and 2250–2300 nm, which diverge from the spectral variations and band sensitivities observed in this study. Zhang et al. [24] found that the NIR regions (990 nm, 1009 nm, 1070 nm, and 1089 nm) were important to LPC estimation for rice by using spectral indices. In the optimal continuous wavelet transform (CWT), the sensitive bands were also 982 nm, 983 nm, 1550 nm, 1679 nm, and 1680 nm. It can be seen that the sensitive bands for LPC are widely distributed, and these differences highlight that different species and cultivars show considerable variation in their responses to phosphorus levels. Notably, in our research under extremely low phosphorus or organic phosphorus conditions, the spectral reflectance responses to phosphorus exhibited nonlinearity, suggesting complex interactions that need further investigation.

Regarding LPC estimation via spectral indices, prior research predominantly relied on single sensitive bands or traditional vegetation indices (e.g., NDVI, RVI) [22]. However, as the vegetation height and coverage increase, these indices tend to saturate, complicating their interaction with spectral data. This study addressed these limitations by calculating two-dimensional optimized spectral indices derived from raw spectra and FOD. Compared with conventional first- and second-order derivatives, FOD demonstrated superior sensitivity in band selection, enhancing the quantification and generalizability of vegetation indices, mitigating collinearity, and providing more accurate data inputs, thereby improving phosphorus prediction accuracy. These findings align with Zhang’s work [24], emphasizing the critical role of band optimization strategies in advancing crop parameter estimation, despite similarities in dual-band combinatorial principles.

Unlike the traditional estimation methods based on empirical models or simple statistical analyses, machine learning methods possess high adaptability and generalization capabilities. In general, the estimation accuracy is affected by crop species, vegetation parameters, spectral indices, and the type of machine learning algorithm. In this study, the RF algorithm was developed to achieve robust performance in estimating LPC in cotton. These results indicate that the models coupled with SI and FOD required significantly fewer input features than the entire original spectrum, yet achieved improved accuracy. This improvement is likely due to the reduction in data dimensionality, which helps eliminate ineffective bands and reduces the autocorrelation caused by large input datasets, thereby enhancing the model’s accuracy and efficiency. This finding aligned with Li et al. [25], who noted that the RF-based LPC estimation model for Citrus Tree had the highest accuracy, compared to the back-propagation neural network (BPNN), partial least squares (PLS), and support vector machine (SVM). But previous studies showed the different performances of various algorithms; for example, Gao’s [17] evaluation of forage phosphorus content estimation using an artificial neural network (ANN), random forest (RF), and support vector machines (SVM), highlighted SVM’s superior predictive capability. This underscores the varied adaptability of machine learning methods in handling complex linear or nonlinear relationships, necessitating further validation.

While this study yielded promising results, several limitations warrant attention. For instance, in this study, the control of external condition stress was limited to the amount of phosphorus application, mainly focusing on the influence of phosphorus on the spectrum. No systematic control or distinction was made for other nutrient elements or stress factors. This is also one of the limitations of this study. The spectrum is a comprehensive manifestation of various physiological, biochemical, and structural characteristics of vegetation leaves. Among these factors, such as the nitrogen content, water conditions, trace element levels, and pests and diseases, all have significant impacts on the spectrum. Especially, the change in nitrogen has a particularly obvious influence on the visible light and near-infrared bands, which may interfere with the identification of phosphorus-related spectral characteristics. There are still challenges in achieving higher-precision LPC monitoring in complex field environments by relying solely on spectral characteristics for single-factor identification. In subsequent studies, common nitrogen application and water volume can also be included in the stress study. Furthermore, the estimation of LPC in this study was limited to the seedling stage under phosphorus toxicity stress. Future research should incorporate full growth stage trials and integrate water and nitrogen stressors to develop universal monitoring models with enhanced agricultural applicability. Additionally, recent advances in deep learning (deep forest conventional neural networks, DP) algorithms have demonstrated exceptional performance in estimating crop nitrogen, water, and chlorophyll contents [26]. However, sample size constraints precluded their application here. Subsequent studies should expand datasets across growth stages, employ three-dimensional spectral indices, and adopt diverse modeling approaches to further refine phosphorus monitoring precision.

## 5. Conclusions

In this study, we integrated the SI and FOD of the original spectrum with RF machine learning algorithms to offer an optimal prediction model for phosphorus content in cotton.

(1) The spectral changes of 24 cotton cultivars are basically consistent.
(2) In the visible region, the reflectance of cotton under phosphorus treatments did not show obvious regularity, while in the NIR, the reflectance of cotton increased with the increase in phosphorus content, showing a certain difference in phosphorus gradient.
(3) The DSI+FOD coupling with the RF model is superior to the other two spectral index models (the NDSI and RSI). This study provides a new perspective to effectively estimate the phosphorus content in cotton leaves. However, spectral data enhancement, the optimization of the random forest algorithm, and the full growth stages of cotton all warrant further investigation.

## Figures and Tables

**Table 1:** Statistical LPC in cotton (mg·g^−1^).

Sample Set	Sample Size	Min	Max	Mean	SD	CV (%)
Calibration	36	0.067	0.12	0.093	0.0135	14.5
Validation	24	0.06	0.107	0.085	0.0153	18.0
Total	60	0.06	0.12	0.090	0.0146	16.2

**Table 2:** Correlation between spectra and LPC.

FOD	|R|	Band (nm)	Band Number	FOD	|R|	Band (nm)	Band Number
0	0.422	973	151	1.1	0.503	459	17
0.1	0.466	941	178	1.2	0.508	459	19
0.2	0.455	973	139	1.3	0.528	838	12
0.3	0.471	940	136	1.4	0.540	838	12
0.4	0.494	940	117	1.5	0.546	838	12
0.5	0.514	940	96	1.6	0.547	838	12
0.6	0.507	917	69	1.7	0.544	838	8
0.7	0.509	904	347	1.8	0.539	838	9
0.8	0.512	838	27	1.9	0.532	838	10
0.9	0.496	838	16	2	0.524	838	9
1	0.483	459	16	/	/	/	/

**Table 3:** Calibration and validation of LPC estimation models (mg·g^−1^).

Index	FOD	Variable (nm)	Calibration		Validation	
			R^2^	RMSE	R^2^	RMSE
NDSI	0	835, 839	0.71	0.008	0.81	0.009
	1	606, 1060				
	1.6	491, 487				
	2	522, 341				
RSI	0	835, 839	0.59	0.011	0.68	0.010
	1	617, 1067				
	1.6	852, 737				
	2	606, 1060				
DSI	0	1062, 1065	0.78	0.008	0.85	0.007
	1	675, 839				
	1.6	839, 452				
	2	839, 452				

## Data Availability

The original contributions presented in this study are included in the article. Further inquiries can be directed to the corresponding author.
